# The effect of breathing hypoxic gas (15% FIO_2_) on physiological and behavioral outcomes during simulated driving in healthy subjects

**DOI:** 10.14814/phy2.15963

**Published:** 2024-03-05

**Authors:** Jaspreet Kaur, Lebbathana Manokaran, Michael Thynne, Mirza M. F. Subhan

**Affiliations:** ^1^ School of Biomedical Sciences, Faculty of Health University of Plymouth Plymouth UK; ^2^ Chest Clinic University Hospitals Plymouth NHS Trust Plymouth UK

**Keywords:** driving behavior, healthy subjects, heart rate variability, hypoxia, hypoxic gas, stimulated driving performance

## Abstract

Hypoxia is mainly caused by cardiopulmonary disease or high‐altitude exposure. We used a driving simulator to investigate whether breathing hypoxic gas influences driving behaviors in healthy subjects. Fifty‐two healthy subjects were recruited in this study, approved by the Science and Engineering Ethical Committee. During simulated driving experiments, driving behaviors, breathing frequency, oxygen saturation (SpO_2_), and heart rate variability (HRV) were analyzed. Each subject had four driving sessions; a 10‐min practice and three 20‐min randomized interventions: normoxic room air (21% FIO_2_) and medical air (21% FIO_2_) and hypoxic air (equal to 15% FIO_2_), analyzed by repeated measures ANOVA. Driving behaviors and HRV frequency domains showed no significant change. Heart rate (HR; *p* < 0.0001), standard deviation of the RR interval (SDRR; *p* = 0.03), short‐term HRV (SD1; *p* < 0.0001), breathing rate (*p* = 0.01), and SpO_2_ (*p* < 0.0001) were all significantly different over the three gas interventions. Pairwise comparisons showed HR increased during hypoxic gas exposure compared to both normoxic interventions, while SDRR, SD1, breathing rate, and SpO_2_ were lower. Breathing hypoxic gas (15% FiO_2_, equivalent to 2710 m altitude) may not have a significant impact on driving behavior in healthy subjects. Furthermore, HRV was negatively affected by hypoxic gas exposure while driving suggesting further research to investigate the impact of breathing hypoxic gas on driving performance for patients with autonomic dysfunction.

## 1 INTRODUCTION

Hypoxia can affect persons exposed to low partial pressures of oxygen and often patients with cardiopulmonary disease. For example, chronic lung disease affects over half a billion people globally with many being hypoxic (Labaki & Han, 2020). Hypoxia is known to affect cognition (Nakata et al., 2017). Driving is dependent on complete cognitive ability. This leads to concerns that those who have hypoxia and are prescribed supplemental oxygen via an oxygen concentrator or cylinder have not been given any guidance from regulatory bodies on whether to use supplemental oxygen while driving, more so if the supplemental oxygen results in below normal SpO_2_ values. There is limited research on the possible effects that breathing hypoxic gas may have on subjects while driving.

Previous investigations in healthy subjects have shown an equal balance of studies for and against the effects of hypoxia on driving behavior (DB) (Bloomfield et al., 2023; Colombo et al., 2005; Sung et al., 2005; Zhang et al., 2022). Due to the small number of studies in healthy subjects, COPD patient studies were also used for comparison (Karakontaki et al., 2013; Orth et al., 2008; Skovhus Prior et al., 2015). All these patient studies showed worse DB than healthy subjects; however, one of these studies only assessed memory and attention without using a driving simulator. Due to the discrepancies in the results of previous work and their methodologies, we have identified a gap which we believe should be investigated.

Hypoxia is also known to activate peripheral chemoreceptors in the sympathetic nervous system (SNS) (Prabhakar et al., 2015). The SNS and the parasympathetic nervous system (PNS) are both part of the autonomic nervous system (ANS). The ANS can be evaluated by heart rate variability (HRV) using an electrocardiogram and has been studied during hypoxia (Zhang et al., 2014).

With regard to the rationale for this study, evidence has shown that COPD patients with hypoxemia underperformed evaluations on driving ability compared to healthy subjects (Karakontaki et al., 2013).

This study aims to investigate whether breathing hypoxic gas influences DB and HRV in healthy subjects using a driving simulator. Along with assessing driving behaviors, data for breathing frequency and oxygen saturation, were also collated.

## 2 SUBJECTS AND METHODS

### 2.1 Participants

Fifty‐two healthy subjects participated in this study and were recruited by convenient sampling. This study was approved by the Science and Engineering Ethical Committee, University of Plymouth and procedures were in accordance with the Declaration of Helsinki. Prior to the experiment, written informed consent was obtained from all participants and an information sheet which outlined their right to withdraw at any point during the experiment was given. Inclusion criteria was healthy subjects aged 18–70 years. Exclusion criteria included subjects with cardiorespiratory or chronic disease, assessed by a questionnaire. Subjects who tested positive for COVID‐19 after undertaking a COVID‐19 rapid lateral flow antigen self‐test issued by the NHS on the day or the night before the experiment, were also not able to participate. Estimating 500 persons with mild hypoxia in our city, and using a confidence interval of 95%, an error of 5% and SD of 0.5 (often used when unaware of the SD), our sample size calculation was 197.

### 2.2 Experimental protocol

**FIGURE 1:**
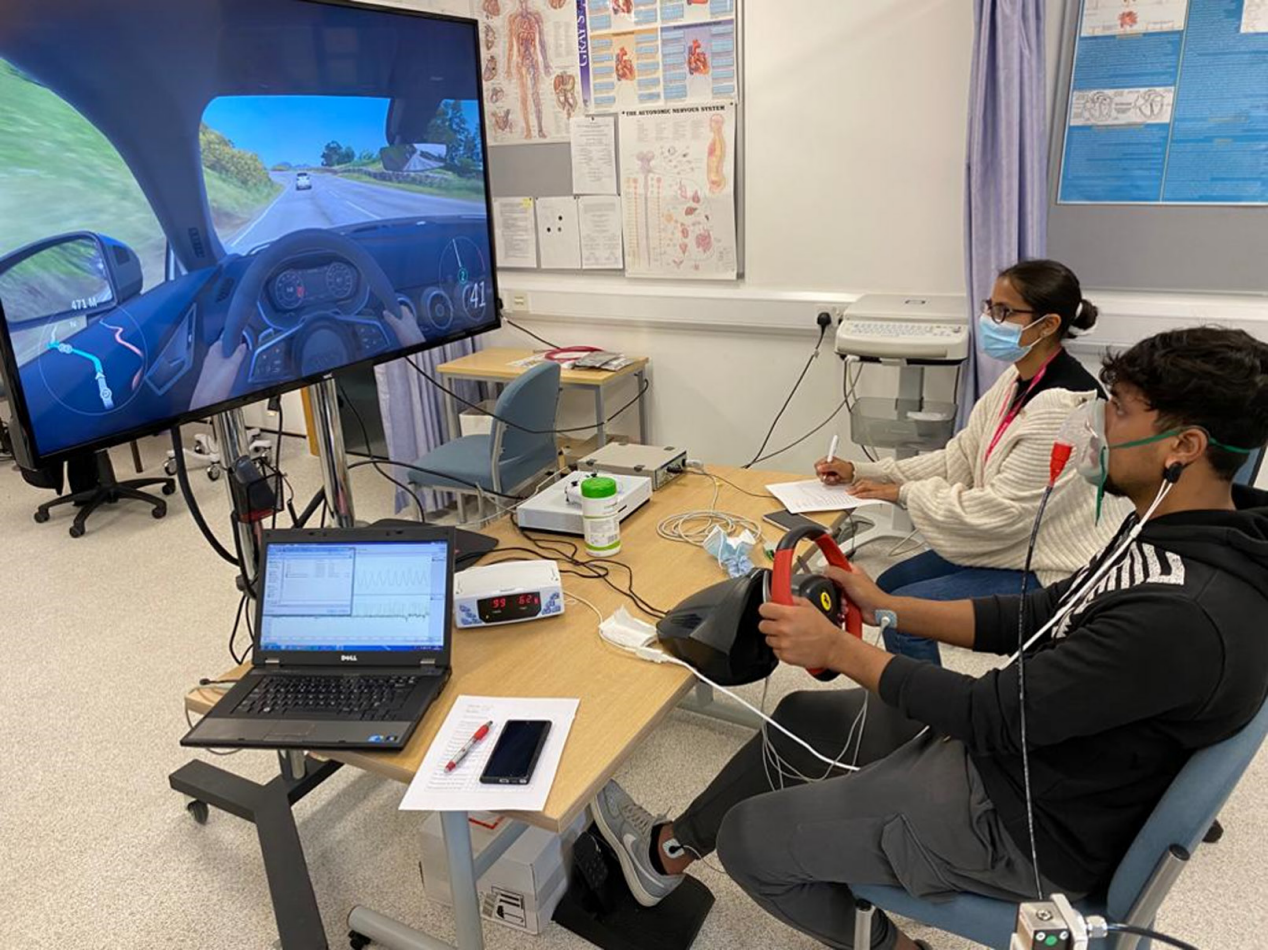
Subject participating in one of the interventions which also shows their view of the Forza Horizon 4‐simulated driving software (permission taken from the subject).

Data regarding their age, sex, ethnicity, and lifestyle habits was collected via a health questionnaire and then had their height (Seca stadiometer), weight (Seca weighing scales), blood pressure, heart rate, and SpO_2_ measured (Welch Allyn Vital Signs Monitor). Then, ECG electrodes, a chest plethysmograph, and a SpO_2_ device were attached to the participant. Driving performance was assessed while participants were seated in front of a 58‐inch TV screen (NEC Display Solutions) which was connected to an Xbox 360 game console (Microsoft). The simulated driving software used was Forza Horizon 4 (Microsoft), which utilized a steering wheel with 2‐foot pedals (Thrustmaster Ferrari 458 Spider; Figure 1). Oxygen saturation, breathlessness, breathing rate, heart rate variability, driving behaviors, and overall preference were measured during simulated driving for each gas intervention.

### 2.3 Simulated driving protocol

Each subject had four driving sessions: a 10‐min practice, 20‐min normoxic room air (21% FIO_2_), 20‐min normoxic medical air (21% FIO_2_), and 20‐min hypoxic air (equal to 15% FIO_2_). Hypoxic gas was given via 100% nitrogen and a 40% Venturi gas mask (Intersurgical EcoLite) (Robson et al., 2000). The level of hypoxia used had an equivalent altitude of 2710 m, close to a PO_2_ of 114 mmHg, and the altitude at which we undertook the study was 18 m. The mask was worn by subjects throughout the three different gas interventions, which were randomized and single blinded, with a rest break of a few minutes in between sessions. Subjects were instructed to drive safely by following The Highway Code (The Department of Transport, 2022). If they were not familiar with The Highway Code, subjects were instructed to drive safely. Subjects were given verbal prompts in the practice session if they were not following The Highway Code. The software has a built‐in satnav, which all subjects used, and a speedometer was clearly visible to all subjects. The route was similar for all interventions. The software also provided rear‐view and left‐side mirrors. All simulated driving sessions were recorded on the hard drive of the game console. Experiments were conducted in a laboratory with the lights turned off and minimal distractions.

### 2.4 Heart rate variability

Heart rate variability (HRV) was assessed using a four‐limb lead electrocardiogram (ECG). Leads were connected to an electrode placed on each wrist and ankle. The leads were connected to data acquisition equipment (PowerLab 26T, ADInstruments) and analyzed by LabChart HRV software (version 8).

### 2.5 Respiratory parameters

SpO_2_ was recorded every minute throughout the experiment using a pulse oximeter (BCI Autocorr Digital Pulse Oximeter) and the change (delta) was analyzed. Breathing rates were analyzed using a chest plethysmograph connected to the data acquisition equipment and software. Breathlessness was estimated using a modified Borg scale from 0 to 10.

### 2.6 Driving behaviors

Driving behaviors (DB) were scored by 13 positive or negative parameters on a chart (Data S1). A positive behavior would include slowing down at a junction/roundabout and negative behavior would include speeding or crossing lanes. For each gas intervention, driving behaviors were assessed by two investigators and a moderated score was given. Discrepancies sometimes occurred as one investigator would be responsible to record SpO_2_ data and regularly check if vital signs were normal (such as heart rate and the ECG). If there were any differences in scoring, the recorded experiment was replayed and re‐checked, although this only occurred in a minor number of occasions. Subject 47 drove in a reckless and erratic manner; therefore, their DB data was excluded.

### 2.7 Statistics

Analysis was not conducted blind to the experimental conditions. LabChart software gave HRV frequency domain data such as low‐frequency normalized units (LFnu), high‐frequency normalized units (HFnu), ratio LF/HF, and time domain data such as heart rate (HR), standard deviation of all the R‐R intervals (SDRR), short‐term HRV (SD1), and long‐term HRV (SD2).

We will refer to this data as non‐normalized, and normalized data was prefixed with “n,” for example, nSDRR. HRV data was adjusted by the heart rate to produce normalized HRV (Sacha et al., 2013) and this was reported in a separate section. The data analysis on HRV is presented in separate sections:

1. Non‐normalized and normalized repeated measures analysis of variance (RMANOVA) 20‐min analysis of the three gas interventions (room air, medical air and hypoxic gas).
2. Non‐normalized and normalized RMANOVA 20‐min analysis of the three gas interventions in the actual chronological order they occurred (to assess a possible repetitive effect).
3. Non‐normalized and normalized paired t‐test analysis of the 0–10 min practice session compared with the 0–10 min room air gas intervention (to assess reproducibility).
4. An unpaired *t*‐test was used for sex and ethnicity comparisons.
5. Stepwise multiple regression analysis was performed with DB as the dependent variable and all other parameters in Tables 1, 2, 3 as independent variables in the model which combined all gas interventions.

Overall, driving behavior scores for each complete driving session for each participant were also statistically analyzed. SPSS (Version 25, IBM) was used for statistical analysis. Means and standard deviation (SD) were used for descriptive analysis. RMANOVA data was tested for sphericity with Mauchly's test and if necessary was corrected using the Greenhouse–Geisser's method and if it was significant, the Bonferroni post hoc test was performed. Significance was taken as *p* < 0.05.

**TABLE 1:** Mean (SD), range and median (interquartile range) age, anthropometric, physiological, and questionnaire data (*n* = 52).

Anthropometric and questionnaire data	Mean (SD)	Range	Median (interquartile range)
Age (years)	22.1 (4.3)	18.0–40.0	21.0 (2.0)
BMI (kg m^−2^)	25.5 (5.6)	18.7–41.9	23.9 (5.2)
SBP (mmHg)	121.9 (12.2)	100.0–155.0	123.0 (19.0)
DBP (mmHg)	73.8 (10.6)	47.0–101.0	74.0 (15.3)
Resting heart rate (beats min^−1^)	77.9 (11.2)	56.0–101.0	77.0 (18.0)
SpO_2_ (%)	98.6 (1.1)	96.0–100.0	99.0 (1.8)
Caffeine (intake per week)	7.7 (9.9)	0.0–40.0	3.5 (12.0)
Exercise (number per week)	3.2 (1.7)	0.0–7.0	3.0 (3.0)
Hours of driving per week	4.2 (10.0)	0.0–60.0	0.0 (3.0)
Penalties points (endorsements) on driving license	0.1 (0.3)	0.0–1.0	0.0 (0.0)
Years of holding a driving license	2.5 (4.2)	0.0–23.0	1.3 (3.0)
Years of racing gaming experience	2.3 (4.8)	0.0–25.0	0.0 (2.0)
Hours of racing gaming per week	2.0 (8.4)	0.0–60.0	0.0 (1.8)

Abbreviations: BMI, body mass index; S and DBP, systolic and diastolic blood pressure; SpO_2_, peripheral oxygen saturation.

**TABLE 2:** Mean (SD) driving behavior for three gas interventions and actual chronological order (*n* = 52).

Variables	Room air	Medical air	Hypoxia (N_2_)	*p* Value
Gas interventions				
Driving behaviors (*n* = 51)	−17.2 (42.7)	−12.5 (33.2)	−21.8 (46.5)	0.28

Variable	1st intervention	2nd intervention	3rd intervention	*p* Value
Actual chronological order				
Driving behaviors (*n* = 51)	−21.6 (49.0)	−13.6 (32.3)	−15.5 (40.8)	0.365

*Note*: Statistical test: repeated measures ANOVA.

**TABLE 3:** Mean (SD) HRV and respiratory data for all three gas interventions (*n* = 52).

Variables	Room air	Medical air	Hypoxia (N_2_)	*p* value
Non‐normalized HRV data				
Heart rate (beats min^−1^)	80.3 (13.3)	80.2 (12.6)	82.7 (13.2)	<0.00001
LFnu (nu)	60.9 (17.2)	63.43 (16.8)	64.1 (17.2)	0.083
HFnu (nu)	33.9 (15.0)	33.4 (14.7)	32.7 (15.6)	0.653
Ratio LF/HF	2.6 (2.4)	2.7 (2.3)	2.8 (2.2)	0.557
SDRR (ms)	54.0 (19.6)	51.6 (17.3)	49.4 (19.4)	0.008
SD1 (ms)	30.4 (15.8)	27.7 (14.2)	24.7 (13.2)	0.00003
SD2 (ms)	69.1 (25.2)	67.0 (21.7)	65.0 (25.1)	0.094
Normalized HRV data				
nLFnu (nu (ms)^−2^)	0.00011 (0.00005)	0.00012 (0.00006)	0.00012 (0.00005)	0.082
nHFnu (nu (ms)^−2^)	0.00006 (0.00003)	0.00006 (0.00003)	0.00006 (0.00003)	0.898
nSDRR	0.069 (0.021)	0.067 (0.019)	0.065 (0.021)	0.102
nSD1	0.039 (0.019)	0.036 (0.017)	0.032 (0.016)	0.0001
nSD2	0.088 (0.028)	0.087 (0.024)	0.085 (0.026)	0.467
Respiratory data				
Breathing rate (breaths min^−1^)	19.5 (3.8)	18.8 (3.5)	18.6 (3.4)	0.005
Absolute SpO_2_ (%)	96.7 (1.5)	96.9 (1.2)	91.7 (2.4)	<0.00001
Delta SpO_2_ (%)	−0.2 (1.2)	−0.2 (0.9)	−3.9 (2.2)	<0.00001

*Note*: Statistical test: repeated measures ANOVA.

## 3 RESULTS

### 3.1 Age, anthropometric, physiological, and questionnaire data

Mean and median age, anthropometric, physiological, and questionnaire data is shown in Table 1 for all 52 subjects. Of the subjects, 24 were females and 28 were males. In terms of ethnicity, 27 Caucasians, 14 Asians, and 11 Afro‐Caribbean subjects took part in this study.

### 3.2 Driving behavior (DB)

#### 3.2.1 DB data of the three gas interventions (room air, medical air, and hypoxic gas)

The mean driving behavior and *p* values for all gas interventions and in their actual chronological order is shown in Table 2. Out of all the gas interventions, hypoxic gas showed the greatest negative effect on driving behaviors, although this was not statistically significant. Driving behavior means had a high SD. Boxplots were used in SPSS to determine mild (>1.5 × inter quartile range) and extreme outliers (>3 × inter quartile range). RMANOVA was performed on two models of outlier removal. The first removed all five extreme outlier subjects (two from room air, one from medical air, and two from hypoxic gas; *p* = 0.22). The second removed eight mild and extreme outlier subjects (four from room air, four from medical air, and six from hypoxic gas; *p* = 0.62); some of these subjects had more than one outlier in the gas interventions. Removal of mild and extreme outliers had no effect on the DB gas intervention results.

#### 3.2.2 DB data in actual chronological order

With chronological order, DB data showed no significant difference over time (Table 2). Upon comparison, a greatest negative driving behavior was seen in the first intervention. Like done with gas intervention data, RMANOVA was performed on two models of outlier removal. The first removed all extreme outliers from six subjects (four from first, two from second, and two from the third intervention; *p* = 0.73). Some subjects had more than one outlier. The second model removed additional mild outliers (from two subjects) along with the extreme ones (two from first, two from second, and one from the third intervention; *p* = 0.94). Removal of mild and extreme outliers had no effect on the DB chronological order results.

#### 3.2.3 Sex and ethnicity DB analysis

**FIGURE 2:**
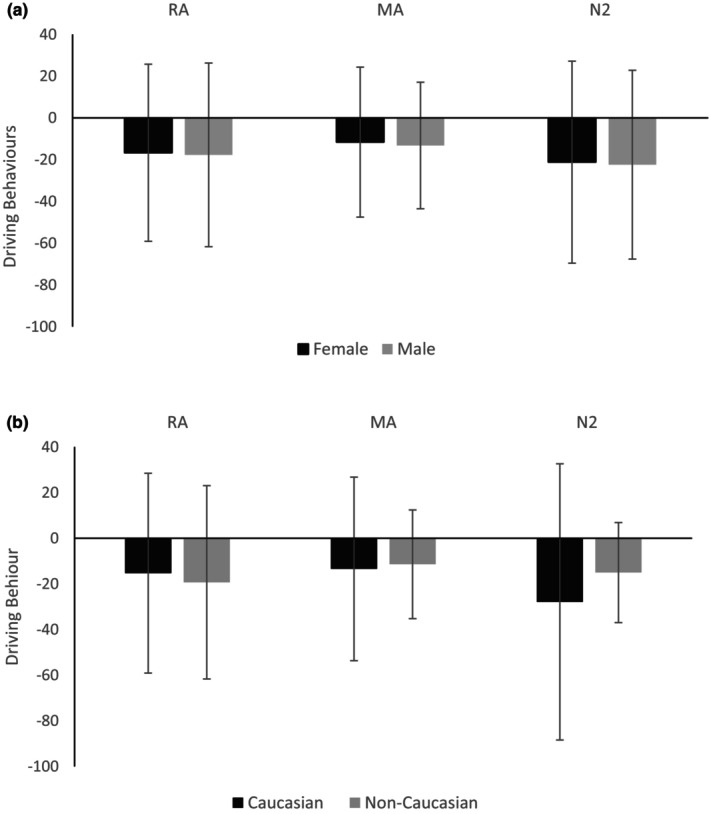
Bar chart showing mean (SD) driving behavior for all three gas interventions based on their (a) sex and (b) ethnicity (*n* = 51). N_2_, Nitrogen; MA, Medical air; RA, Room air.

DB for all three interventions showed no significant difference when analyzed for sex (Figure 2a; female vs. male) and ethnicity (Figure 2b; Caucasian vs. non‐Caucasian).

### 3.3 HRV and respiratory data

#### 3.3.1 HRV and respiratory data analysis of the three gas interventions

Heart rate was significantly higher over the three interventions (Table 3) and pairwise comparison showed it was higher during hypoxic gas compared to room air (*p* = 0.00006) and medical air (*p* = 0.00002). LFnu, HFnu, LF/HF ratio, SD2, nLFnu, nHFnu, nSD2, and nSDRR did not show any significant change. SDRR was significantly different with hypoxic gas and pairwise comparison showed it was lower for hypoxic gas relative to room air (*p* = 0.028). Similarly, both SD1 and nSD1 showed a significant difference across interventions. Pairwise comparison for SD1 showed hypoxic gas was lower than room air (*p* = 0.0002) and medical air (*p* = 0.04). Data for room air were also lower compared to medical air (*p* = 0.04). Pairwise comparisons for nSD1 were similar.

Breathing rates were significantly different and pairwise comparisons showed they were significantly higher for room air when compared with medical air (*p* = 0.02) and hypoxic gas (*p* = 0.02). Delta and absolute SpO_2_ indicated hypoxic gas resulted in a significant desaturation, and pairwise results showed it was lower than room (*p* < 0.00001) and medical air (*p* < 0.00001). Nine subjects had a SpO_2_ <89% (17% of all subjects).

#### 3.3.2 HRV and respiratory data analysis in actual chronological order

Heart rate was significantly lower over the three interventions (Table 4) and pairwise comparison showed it was lower during the third intervention compared to the first (*p* = 0.0006) and second (*p* = 0.016). LFnu, HFnu, LF/HF ratio, SD1, nLFnu, nSD1, nSD2, and nSDRR did not show any significant differences. SDRR was significantly different over time and pairwise comparison showed it was higher for the third intervention compared to the second (*p* = 0.012). SD2 was also significantly different over time. Pairwise comparison showed it was higher for the third intervention compared to the first (*p* = 0.027) and second (*p* = 0.007). nHFnu showed a significant decrease over time and pairwise comparison showed it was lower for the third intervention compared to the first (*p* = 0.023).

**TABLE 4:** Mean (SD) HRV and respiratory data in their actual chronological order (*n* = 52).

Variables	First intervention	Second intervention	Third intervention	*p* Value
Non‐normalized HRV data				
Heart rate (beats min^−1^)	82.2 (13.1)	81.2 (13.0)	80.0 (13.1)	0.00008
LFnu (nu)	61.2 (16.5)	63.4 (17.1)	64.4 (17.1)	0.093
HFnu (nu)	34.1 (14.5)	32.7 (15.1)	32.9 (15.4)	0.471
Ratio LF/HF	2.6 (2.6)	2.7 (2.0)	2.8 (2.3)	0.602
SDRR (ms)	50.1 (17.8)	50.6 (18.7)	54.1 (19.9)	0.021
SD1 (ms)	27.0 (14.1)	26.8 (13.6)	28.4 (15.9)	0.349
SD2 (ms)	64.7 (23.1)	66.0 (24.0)	70.6 (24.8)	0.007
Normalized HRV data				
nLFnu (nu (ms)^−2^)	0.00012 (0.00005)	0.00012 (0.00005)	0.00011 (0.00006)	0.592
nHFnu (nu (ms)^−2^)	0.00006 (0.00003)	0.00006 (0.00003)	0.00005 (0.00002)	0.008
nSDRR	0.066 (0.021)	0.066 (0.019)	0.068 (0.020)	0.397
nSD1	0.036 (0.018)	0.035 (0.015)	0.036 (0.018)	0.710
nSD2	0.085 (0.027)	0.086 (0.025)	0.089 (0.025)	0.243
Respiratory data				
Breathing rate (breaths min^−1^)	19.1 (3.7)	19.3 (3.5)	18.4 (3.8)	0.011
Absolute SpO_2_ (%)	95.0 (3.1)	94.6 (3.3)	95.6 (2.5)	0.279
Delta SpO_2_ (%)	−1.3 (2.2)	−1.7 (2.7)	−1.1 (2.1)	0.458

*Note*: Statistical test: repeated measures ANOVA.

The mean and *p*‐value for breathing rate, absolute SpO_2_, and delta SpO_2_ for the three interventions in their actual chronological order is shown in Table 4. Over time, there was a significant decrease in breathing rate; pairwise comparison showed the third intervention to be lower than the second (*p* = 0.003). Absolute and delta SpO_2_ data showed no significant difference over time.

### 3.4 Reproducibility

#### 3.4.1 HRV and respiratory data analysis of the 0–10 min practice session compared with the 0–10 min room air gas intervention

The means and *p*‐values of HRV and respiratory data for the first 10 min of practice and room air are shown in Table 5. Heart rate (*p* < 0.00001), nLFnu (*p* = 0.022), and breathing rate (*p* = 0.008) were significantly lower for room air compared to practice. All other HRV variables, absolute and delta SpO_2_ were not significantly different.

**TABLE 5:** Mean (SD) HRV and respiratory data for first 10 min of both practice and room air interventions (*n* = 52).

Variables	Practice	Room air	% difference	*p* value
Non‐normalized HRV data				
Heart rate (beats min^−1^)	83.1 (13.9)	79.8 (12.8)	−4.0	<0.00001
LFnu (nu)	56.8 (17.4)	59.1 (19.4)	4.0	0.184
HFnu (nu)	36.0 (13.5)	34.4 (16.5)	−4.4	0.366
Ratio LF/HF	2.2 (2.4)	2.6 (2.6)	18.2	0.061
SDRR (ms)	54.2 (18.0)	54.2 (19.7)	0.0	0.992
SD1 (ms)	30.9 (18.1)	31.9 (18.3)	3.2	0.536
SD2 (ms)	68.3 (21.8)	68.9 (23.6)	0.9	0.825
Normalized HRV data				
nLFnu (nu (ms)^−2^)	0.00013 (0.00007)	0.00011 (0.00005)	−15.4	0.022
nHFnu (nu (ms)^−2^)	0.00006 (0.00003)	0.00006 (0.00003)	0.0	0.261
nSDRR	0.092 (0.131)	0.071 (0.026)	−22.8	0.250
nSD1	0.039 (0.027)	0.042 (0.027)	7.7	0.412
nSD2	0.089 (0.030)	0.090 (0.029)	1.1	0.885
Respiratory data				
Breathing rate (breaths min^−1^)	20.9 (3.3)	19.8 (4.1)	−5.3	0.008
Absolute SpO_2_ (%)	97.0 (1.3)	96.8 (1.3)	−0.2	0.279
Delta SpO_2_ (%)	−0.1 (0.8)	−0.1 (1.0)	0.0	0.790

*Note*: Statistical test: paired *t*‐test.

A secondary analysis was also conducted on a subset of 17 subjects who performed the room air intervention first; a comparison was made with their own practice data. Paired t‐testing showed only nLFnu was lower for room air compared to practice (*p* = 0.048), all other HRV and respiratory variables were not significantly different.

### 3.5 Regression analysis

**FIGURE 3:**
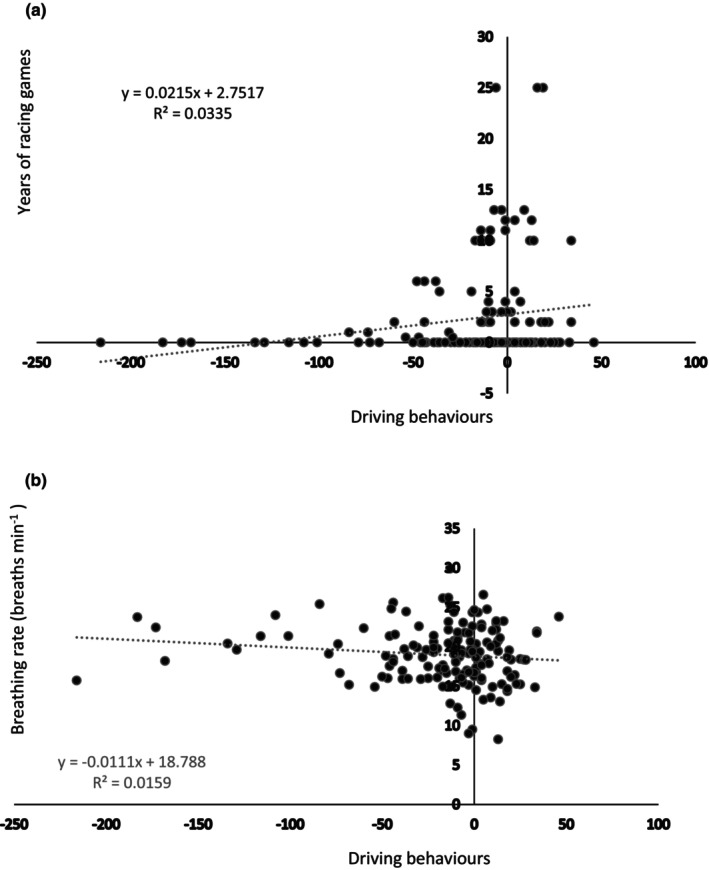
Linear regression of (a) years of racing gaming and (b) breathing rate against driving behaviors for all three gas interventions (*n* = 153; as three data points for all 51 subjects).

Multiple linear regression was carried out with DB as the dependent variable. Age, anthropometric, physiological, and questionnaire data (shown in Table 1), breathing rate, delta SpO_2_, and all HRV variables (shown in Table 3) were independent variables in the model. Regression analysis showed exercise (*p* = 0.001; *r* ^2^ = 0.05) and breathing rate (*p* = 0.045; *r* ^2^ = 0.02) both had a weak negative relationship with DB, while caffeine (*p* = 0.002; *r* ^2^ = 0.06) and years of racing gaming experience (*p* = 0.033; *r* ^2^ = 0.03) both showed a weak positive relationship. Two of these regressions are illustrated below (Figure 3).

## 4 DISCUSSION

An important finding from this study was that DB did not show any significant changes neither in the three gas interventions nor in the actual order. During hypoxic‐simulated driving, heart rate increased while SDRR, breathing rate, and SpO_2_ decreased. When the data were analyzed in actual chronological order, it was seen that breathing rate, heart rate, SDRR, and nHFnu were significantly lower after the third test. DB, SpO_2_, and most HRV variables showed good reproducibility when comparing practice and room air interventions.

Breathing rate showed decreases with both medical air and hypoxic gas. Previous work has shown increases in tidal volume (Mahutte & Rebuck, 1978) and ventilation (Weil & Zwillich, 1976) in healthy subjects during hypoxia which can explain our decrease in rate. However, it is unclear why the rate decreased in medical air. This could be a placebo effect. SpO_2_ was lower with hypoxic gas, as seen previously (Ravi & Subhan, 2023).

DB was a major outcome of our study. There is limited published data investigating the effects of hypoxia on DB during simulated driving in healthy participants. Due to this limitation, patient studies with a similar aim have also been compared. Although our findings were negative, these results have important ramifications for health‐care workers, policymakers, and persons living at high altitude. The mild hypoxic gas used in our study had an equivalent altitude of 2710 m, close to that of Bogota, Columbia. DB scores were used to evaluate our subjects' driving performance in differing gas interventions. Our results showed no significant effect of hypoxic gas on DB; however, the absolute change was worse for hypoxic gas compared to the other two gas interventions. Previous findings in healthy participants have also shown no effect of hypoxia on DB (Colombo et al., 2005; Sung et al., 2005); however, both studies had a low statistical power and only used a few markers of DB, compared to this study. A study showed significant worsening of some driving behaviors at higher altitude (Zhang et al., 2022), although most DB tests focused on eye movements. Unlike this study, which assessed DB continuously throughout the three 20‐min interventions of simulated driving, their study looked at nine time point‐specific hazards. Only one of these studies gave the FIO_2_ concentration. Recently, a study has shown worse driving performance with severe hypoxia; however, this was not surprising as the subjects' mean SpO_2_ was approximately 71% (Bloomfield et al., 2023). We felt their methods of assessing DB and ours had a similar approach.

Three COPD patient studies have shown worse DB than healthy participants (Karakontaki et al., 2013; Orth et al., 2008; Skovhus Prior et al., 2015). The former study found no difference in concentration but did find patients had more accidents than healthy controls. One study did not use a driving simulator and their tests did not necessarily measure DB, but rather cognition, involving memory and attention (Karakontaki et al., 2013). Cognition has been seen to be impaired in COPD, including memory and attention (Torres‐Sanchez et al., 2015). Orth et al. (2008) showed no difference in concentration faults but did show worse accident numbers in COPD compared to controls during simulated driving. COPD patients undergoing long‐term oxygen therapy (LTOT) did worse in driving performance than non‐LTOT COPD patients and healthy subjects, although arterial blood gas data was not provided, so it is difficult to judge if the LTOT patients were more hypoxic than non‐LTOT patients (Skovhus Prior et al., 2015).

Although these few previous studies in healthy subjects and patients have successfully measured driving performance, we do believe our scoring method is a practical and more robust method of assessing DB/driving performance, which others could use in future. This form of assessment is evident in a recent publication (Bloomfield et al., 2023).

Previous work (Vento et al., 2022) supports our finding that biological sex has no effect on cognition with hypoxic gas; however, other normoxic work has shown sex differences in DB (Ferrante et al., 2020). Our data also showed no effect of ethnicity on DB and this has been supported by a survey investigating ethnicity and driving behaviors (Bergdahl, 2007).

Our study showed HR was highest during hypoxic gas compared to the other two gas interventions. Hypoxic gas also resulted in a decrease in SDRR and SD1. Our previous work has also shown an increase HR (Ravi & Subhan, 2023), although it did not show a decrease in SDRR and SD1 with hypoxic gas. One reason for this discrepancy could be a lower statistical power in our previous study. A systematic review of HRV and hypoxia in healthy subjects (Oliveira et al., 2017) has shown SDRR decrease with hypoxia in three studies; however, two other studies showed no change with hypoxia. Reasons for these discrepancies include varied length of hypoxia exposure and differing FIO_2_ concentrations. It is postulated that HR increases during hypoxia due to increased baroreceptor sensitivity (Fox et al., 2006).

The effect of actual chronological order showed BR, HR, nHFnu was lowest in the third test, while SDRR and SD2 were highest. Falls in BR and HR were not surprising as our subjects might have had an initial “white‐coat” effect, which is known to be caused by sympathetic activation (Pioli et al., 2018). Our previous work investigating repeat HRV testing after three tests has shown similar results for HR, nHFnu, and SD2 (Subhan et al., 2015). SDRR also increased but did not reach statistical significance. Interestingly, this previous work also found an increase in LFnu after three tests, while it was also higher in our current one, albeit not significant. It is unclear why nHFnu (parasympathetic) fell after repeat testing, as we would have expected the opposite. This data from our study did include varying interventions, which could be a reason for this unpredicted finding.

Reproducibility was assessed by comparing the first 10 min of our practice and room air data. Unsurprisingly, BR, HR, and nLFnu were lower during RA, most likely due to sympathetic activation being high in the practice session, as mentioned in the paragraph above.

Our regression data indicated that more racing gaming experience and caffeine, resulted in better DB. Both were expected, with more racing gaming improving their driving skills, while caffeine has been seen to improve simulated driving and attention (Brice & Smith, 2001). Additionally, lower BR and exercise frequency, showed better DB. A lower BR could indicate a more relaxed state. It is unclear how exercise frequency was related to DB. In general, causal inference should not be made with correlations. Future studies could investigate these factors in more detail.

Some of our limitations included the finding that breathing hypoxic gas had no significant impact on DB, which could be due to the level of hypoxia experienced by participants, with FiO_2_ not being low enough. However, our SpO_2_ levels (91.7%) were slightly lower to those found in a recent study of COPD patients, where most had a SpO_2_ value between 93% and 96% (Echevarria et al., 2021). Our previous work has shown 12% FIO_2_ can lower cerebral tissue oxygenation (Ravi & Subhan, 2023), so it is unlikely that our subjects had cerebral hypoxia, although one third of our subjects reported drowsiness, less concentration, lethargy, and headaches. Our subjects had varied driving and gaming experiences; however, all were given a practice and as overall mean differences were compared, individual variations would be negligible. Due to logistical constraints, our interventions were 20 min long. It is possible that a longer time period could have a more sustained hypoxic effect. Another issue was the PowerLab equipment was connected through cables, and this did result in some movement artifact effects. Newer wireless technology can overcome this. Another limitation was that we could not reach the predicted sample size estimation. Finally, the assessment of DBs was done manually by two investigators and was subjective. This could lead to differences, but scoring was moderated, resulting in a fair test.

## 5 CONCLUSION

This study indicates that breathing mild hypoxic gas does not have an impact on driving performance in healthy subjects. Although the findings were mostly nonsignificant, this study did look at an important area for road safety, which is relevant to high altitude drivers and possibly hypoxic patients who drive. Therefore, policymakers should be aware that it is unlikely that road safety will be impeded by allowing moderately hypoxic persons to drive. Heart rate variability was affected by breathing hypoxic gas while driving and provides a starting point for conducting further research on the impact hypoxic gas may have on driving performance for patients with autonomic dysfunction. Another important outcome of this study is the development of a robust method to practically assess driving behaviors in different populations.

## AUTHOR CONTRIBUTIONS

Conception or design of the work (JK, LM, MT, and MMFS). Acquisition, analysis, or interpretation of data for the work (JK, LM, and MMFS). Drafting of the work or revising it critically for important intellectual content (JK, LM, MT, and MMFS). We can confirm that all authors (JK, LM, MT, and MMFS) approved the final version of this article, agreed to be accountable for all aspects of the work in ensuring that questions related to the accuracy or integrity of any part of the work are appropriately investigated and resolved and that all persons designated as authors qualify for authorship, and all those who qualify for authorship are listed.

## FUNDING INFORMATION

The authors received no grant funding for this work.

## CONFLICT OF INTEREST STATEMENT

The authors declare that they have no conflict of interest.

## ETHICS STATEMENT

Informed consent was obtained from all subjects. All procedures performed in studies involving human participants were in accordance with the ethical standards of the 1964 Helsinki Declaration and its later amendments or comparable ethical standards. This study was approved by the Faculty of Science and Engineering Research Ethics Committee University of Plymouth.

## Supporting information


Data S1

Data S2

## Data Availability

The data that support the findings of this study are available from the corresponding author upon reasonable request.
