# Supplementary material for: The effect of breathing hypoxic gas (15% FIO2) on physiological and behavioral outcomes during simulated driving in healthy subjects
Source: Physiol Rep. 2024 Mar 5;12(5):e15963. doi: 10.14814/phy2.15963 (PMC10912923; doi:10.14814/phy2.15963)
Supplement: Supplementary file 1 — Data S1 [file PHY2-12-e15963-s001.docx]

Supplementary data 1

**Subject No.:**

Every subject will start at 0.

| Driving behaviours |  | Practice | 3 (RmAir) | 2 (MedAir) | 1 (N2) |
| --- | --- | --- | --- | --- | --- |
| Order |  | N/A |  |  |  |
| Minor collision with another car or surroundings | -5 |  |  |  |  |
| Major collision with another car or surroundings | -10 |  |  |  |  |
| Brief/corrected lane crossing | -1 |  |  |  |  |
| Prolonged lane crossing (>3s) | -3 |  |  |  |  |
| Giving way to overtaking cars | +2 |  |  |  |  |
| Not giving way to overtaking cars | -2 |  |  |  |  |
| Avoiding collision when not at fault | +5 |  |  |  |  |
| Slowing down at junction/roundabout to <30mph | +3 |  |  |  |  |
| Not slowing down at junction/roundabout | -3 |  |  |  |  |
| 10% speeding >33, 44, 55 mph | -1 |  |  |  |  |
| 20% speeding >36, 48, 60 mph | -3 |  |  |  |  |
| 30% speeding >42, 52, 65 mph | -5 |  |  |  |  |
| Dangerous driving/overtaking | -3 |  |  |  |  |
