# Supplementary material for: The effect of breathing hypoxic gas (15% FIO2) on physiological and behavioral outcomes during simulated driving in healthy subjects
Source: Physiol Rep. 2024 Mar 5;12(5):e15963. doi: 10.14814/phy2.15963 (PMC10912923; doi:10.14814/phy2.15963)
Supplement: Supplementary file 2 — Data S2 [file PHY2-12-e15963-s002.docx]

GET

FILE='C:\Users\msubhan\OneDrive - University of Plymouth\2022 projects\Jaspreet summer bursary\Manuscript\SPSS results and data\RMANOVA data regression all interventions n=153.sav'.

DATASET NAME DataSet1 WINDOW=FRONT.

REGRESSION

/MISSING LISTWISE

/STATISTICS COEFF OUTS R ANOVA

/CRITERIA=PIN(.05) POUT(.10)

/NOORIGIN

/DEPENDENT DB1

/METHOD=STEPWISE Age Exercise Alcohol Caff driveweek yearslicense yearsgame hoursgame BMI SBP DBP

RestHR RestSpO2 HR1 LFnu1 HFnu1 Ratio1 SDRR1 SD11 SD21 nLFnu1 nHFnu1 nSD11 nSD21 nSDRR1 BR1

deltaSpO21

/SAVE ZRESID.

**Regression**

| **Notes** | | |
| --- | --- | --- |
| Output Created | | 27-APR-2023 17:05:51 |
| Comments | |  |
| Input | Data | C:\Users\msubhan\OneDrive - University of Plymouth\2022 projects\Jaspreet summer bursary\Manuscript\SPSS results and data\RMANOVA data regression all interventions n=153.sav |
|  | Active Dataset | DataSet1 |
|  | Filter | <none> |
|  | Weight | <none> |
|  | Split File | <none> |
|  | N of Rows in Working Data File | 153 |
| Missing Value Handling | Definition of Missing | User-defined missing values are treated as missing. |
|  | Cases Used | Statistics are based on cases with no missing values for any variable used. |
| Syntax | | REGRESSION  /MISSING LISTWISE  /STATISTICS COEFF OUTS R ANOVA  /CRITERIA=PIN(.05) POUT(.10)  /NOORIGIN  /DEPENDENT DB1  /METHOD=STEPWISE Age Exercise Alcohol Caff driveweek yearslicense yearsgame hoursgame BMI SBP DBP  RestHR RestSpO2 HR1 LFnu1 HFnu1 Ratio1 SDRR1 SD11 SD21 nLFnu1 nHFnu1 nSD11 nSD21 nSDRR1 BR1  deltaSpO21  /SAVE ZRESID. |
| Resources | Processor Time | 00:00:00.03 |
|  | Elapsed Time | 00:00:00.03 |
|  | Memory Required | 41136 bytes |
|  | Additional Memory Required for Residual Plots | 0 bytes |
| Variables Created or Modified | ZRE_8 | Standardized Residual |

[DataSet1] C:\Users\msubhan\OneDrive - University of Plymouth\2022 projects\Jaspreet summer bursary\Manuscript\SPSS results and data\RMANOVA data regression all interventions n=153.sav

| **Variables Entered/Removed^a^** | | | |
| --- | --- | --- | --- |
| Model | Variables Entered | Variables Removed | Method |
| 1 | Caff | . | Stepwise (Criteria: Probability-of-F-to-enter <= .050, Probability-of-F-to-remove >= .100). |
| 2 | Exercise | . | Stepwise (Criteria: Probability-of-F-to-enter <= .050, Probability-of-F-to-remove >= .100). |
| 3 | BR1 | . | Stepwise (Criteria: Probability-of-F-to-enter <= .050, Probability-of-F-to-remove >= .100). |
| 4 | yearsgame | . | Stepwise (Criteria: Probability-of-F-to-enter <= .050, Probability-of-F-to-remove >= .100). |
| a. Dependent Variable: DB1 | | | |

| **Model Summary^e^** | | | | |
| --- | --- | --- | --- | --- |
| Model | R | R Square | Adjusted R Square | Std. Error of the Estimate |
| 1 | .253^a^ | .064 | .058 | 39.94366 |
| 2 | .360^b^ | .130 | .118 | 38.65091 |
| 3 | .391^c^ | .153 | .136 | 38.25777 |
| 4 | .423^d^ | .179 | .157 | 37.79725 |
| a. Predictors: (Constant), Caff | | | | |
| b. Predictors: (Constant), Caff, Exercise | | | | |
| c. Predictors: (Constant), Caff, Exercise, BR1 | | | | |
| d. Predictors: (Constant), Caff, Exercise, BR1, yearsgame | | | | |
| e. Dependent Variable: DB1 | | | | |

| **ANOVA^a^** | | | | | | |
| --- | --- | --- | --- | --- | --- | --- |
| Model | | Sum of Squares | df | Mean Square | F | Sig. |
| 1 | Regression | 16431.719 | 1 | 16431.719 | 10.299 | .002^b^ |
|  | Residual | 239324.379 | 150 | 1595.496 |  |  |
|  | Total | 255756.099 | 151 |  |  |  |
| 2 | Regression | 33166.047 | 2 | 16583.023 | 11.101 | .000^c^ |
|  | Residual | 222590.052 | 149 | 1493.893 |  |  |
|  | Total | 255756.099 | 151 |  |  |  |
| 3 | Regression | 39134.862 | 3 | 13044.954 | 8.913 | .000^d^ |
|  | Residual | 216621.237 | 148 | 1463.657 |  |  |
|  | Total | 255756.099 | 151 |  |  |  |
| 4 | Regression | 45747.139 | 4 | 11436.785 | 8.005 | .000^e^ |
|  | Residual | 210008.959 | 147 | 1428.632 |  |  |
|  | Total | 255756.099 | 151 |  |  |  |
| a. Dependent Variable: DB1 | | | | | | |
| b. Predictors: (Constant), Caff | | | | | | |
| c. Predictors: (Constant), Caff, Exercise | | | | | | |
| d. Predictors: (Constant), Caff, Exercise, BR1 | | | | | | |
| e. Predictors: (Constant), Caff, Exercise, BR1, yearsgame | | | | | | |

| **Coefficients^a^** | | | | | | |
| --- | --- | --- | --- | --- | --- | --- |
| Model | | Unstandardized Coefficients | | Standardized Coefficients | t | Sig. |
|  |  | B | Std. Error | Beta |  |  |
| 1 | (Constant) | -25.370 | 4.085 |  | -6.211 | .000 |
|  | Caff | 1.067 | .333 | .253 | 3.209 | .002 |
| 2 | (Constant) | -6.358 | 6.920 |  | -.919 | .360 |
|  | Caff | 1.172 | .323 | .278 | 3.624 | .000 |
|  | Exercise | -6.134 | 1.833 | -.257 | -3.347 | .001 |
| 3 | (Constant) | 29.676 | 19.113 |  | 1.553 | .123 |
|  | Caff | 1.148 | .320 | .273 | 3.585 | .000 |
|  | Exercise | -6.796 | 1.843 | -.285 | -3.686 | .000 |
|  | BR1 | -1.773 | .878 | -.155 | -2.019 | .045 |
| 4 | (Constant) | 35.945 | 19.107 |  | 1.881 | .062 |
|  | Caff | 1.019 | .322 | .242 | 3.163 | .002 |
|  | Exercise | -6.681 | 1.822 | -.280 | -3.666 | .000 |
|  | BR1 | -2.248 | .895 | -.197 | -2.512 | .013 |
|  | yearsgame | 1.445 | .672 | .169 | 2.151 | .033 |
| a. Dependent Variable: DB1 | | | | | | |

| **Excluded Variables^a^** | | | | | | |
| --- | --- | --- | --- | --- | --- | --- |
| Model | | Beta In | t | Sig. | Partial Correlation | Collinearity Statistics |
|  |  |  |  |  |  | Tolerance |
| 1 | Age | -.037^b^ | -.398 | .691 | -.033 | .739 |
|  | Exercise | -.257^b^ | -3.347 | .001 | -.264 | .991 |
|  | Alcohol | .033^b^ | .395 | .694 | .032 | .906 |
|  | driveweek | .052^b^ | .629 | .530 | .051 | .917 |
|  | yearslicense | .014^b^ | .146 | .884 | .012 | .639 |
|  | yearsgame | .138^b^ | 1.740 | .084 | .141 | .972 |
|  | hoursgame | .036^b^ | .446 | .656 | .037 | .986 |
|  | BMI | .125^b^ | 1.585 | .115 | .129 | .998 |
|  | SBP | .065^b^ | .816 | .416 | .067 | 1.000 |
|  | DBP | .098^b^ | 1.200 | .232 | .098 | .940 |
|  | RestHR | .050^b^ | .626 | .532 | .051 | .998 |
|  | RestSpO2 | .050^b^ | .620 | .536 | .051 | .980 |
|  | HR1 | .057^b^ | .714 | .476 | .058 | .994 |
|  | LFnu1 | .005^b^ | .064 | .949 | .005 | .986 |
|  | HFnu1 | .023^b^ | .288 | .774 | .024 | .989 |
|  | Ratio1 | .003^b^ | .037 | .970 | .003 | .976 |
|  | SDRR1 | .022^b^ | .273 | .785 | .022 | .997 |
|  | SD11 | -.010^b^ | -.128 | .898 | -.011 | .999 |
|  | SD21 | .031^b^ | .387 | .699 | .032 | .998 |
|  | nLFnu1 | .027^b^ | .344 | .731 | .028 | .999 |
|  | nHFnu1 | .033^b^ | .411 | .681 | .034 | .972 |
|  | nSD11 | .003^b^ | .034 | .973 | .003 | .999 |
|  | nSD21 | .066^b^ | .830 | .408 | .068 | 1.000 |
|  | nSDRR1 | .053^b^ | .672 | .503 | .055 | 1.000 |
|  | BR1 | -.105^b^ | -1.331 | .185 | -.108 | .997 |
|  | deltaSpO21 | -.011^b^ | -.134 | .894 | -.011 | .990 |
| 2 | Age | -.017^c^ | -.194 | .846 | -.016 | .736 |
|  | Alcohol | .011^c^ | .131 | .896 | .011 | .899 |
|  | driveweek | -.005^c^ | -.058 | .954 | -.005 | .876 |
|  | yearslicense | .031^c^ | .324 | .746 | .027 | .637 |
|  | yearsgame | .120^c^ | 1.552 | .123 | .127 | .967 |
|  | hoursgame | .018^c^ | .227 | .821 | .019 | .981 |
|  | BMI | .121^c^ | 1.593 | .113 | .130 | .997 |
|  | SBP | .054^c^ | .702 | .484 | .058 | .998 |
|  | DBP | .048^c^ | .599 | .550 | .049 | .905 |
|  | RestHR | .004^c^ | .053 | .958 | .004 | .967 |
|  | RestSpO2 | .020^c^ | .253 | .801 | .021 | .966 |
|  | HR1 | .002^c^ | .024 | .981 | .002 | .949 |
|  | LFnu1 | .038^c^ | .494 | .622 | .041 | .970 |
|  | HFnu1 | .010^c^ | .129 | .898 | .011 | .986 |
|  | Ratio1 | -.004^c^ | -.052 | .959 | -.004 | .976 |
|  | SDRR1 | .048^c^ | .621 | .535 | .051 | .987 |
|  | SD11 | -.005^c^ | -.063 | .950 | -.005 | .999 |
|  | SD21 | .065^c^ | .847 | .399 | .069 | .981 |
|  | nLFnu1 | .008^c^ | .099 | .922 | .008 | .993 |
|  | nHFnu1 | -.016^c^ | -.200 | .842 | -.016 | .938 |
|  | nSD11 | -.014^c^ | -.188 | .851 | -.015 | .995 |
|  | nSD21 | .075^c^ | .982 | .328 | .080 | .999 |
|  | nSDRR1 | .052^c^ | .682 | .496 | .056 | 1.000 |
|  | BR1 | -.155^c^ | -2.019 | .045 | -.164 | .966 |
|  | deltaSpO21 | -.019^c^ | -.242 | .809 | -.020 | .989 |
| 3 | Age | -.020^d^ | -.224 | .823 | -.018 | .736 |
|  | Alcohol | .015^d^ | .183 | .855 | .015 | .899 |
|  | driveweek | -.033^d^ | -.403 | .688 | -.033 | .851 |
|  | yearslicense | .019^d^ | .199 | .842 | .016 | .634 |
|  | yearsgame | .169^d^ | 2.151 | .033 | .175 | .908 |
|  | hoursgame | .051^d^ | .651 | .516 | .054 | .941 |
|  | BMI | .106^d^ | 1.397 | .165 | .114 | .986 |
|  | SBP | .011^d^ | .135 | .893 | .011 | .916 |
|  | DBP | .036^d^ | .455 | .650 | .037 | .900 |
|  | RestHR | .031^d^ | .396 | .693 | .033 | .940 |
|  | RestSpO2 | .039^d^ | .498 | .620 | .041 | .953 |
|  | HR1 | .020^d^ | .249 | .803 | .021 | .937 |
|  | LFnu1 | .036^d^ | .464 | .643 | .038 | .970 |
|  | HFnu1 | -.011^d^ | -.144 | .886 | -.012 | .968 |
|  | Ratio1 | -.005^d^ | -.062 | .951 | -.005 | .976 |
|  | SDRR1 | -.024^d^ | -.277 | .782 | -.023 | .800 |
|  | SD11 | -.048^d^ | -.614 | .540 | -.051 | .930 |
|  | SD21 | -.006^d^ | -.068 | .946 | -.006 | .778 |
|  | nLFnu1 | .021^d^ | .275 | .783 | .023 | .985 |
|  | nHFnu1 | -.021^d^ | -.265 | .791 | -.022 | .937 |
|  | nSD11 | -.045^d^ | -.575 | .566 | -.047 | .960 |
|  | nSD21 | .019^d^ | .226 | .822 | .019 | .848 |
|  | nSDRR1 | -.005^d^ | -.061 | .952 | -.005 | .863 |
|  | deltaSpO21 | -.009^d^ | -.120 | .904 | -.010 | .985 |
| 4 | Age | -.048^e^ | -.540 | .590 | -.045 | .721 |
|  | Alcohol | -.099^e^ | -1.081 | .281 | -.089 | .659 |
|  | driveweek | -.035^e^ | -.429 | .669 | -.035 | .851 |
|  | yearslicense | -.039^e^ | -.398 | .691 | -.033 | .587 |
|  | hoursgame | .048^e^ | .617 | .538 | .051 | .940 |
|  | BMI | .098^e^ | 1.302 | .195 | .107 | .983 |
|  | SBP | -.006^e^ | -.076 | .939 | -.006 | .907 |
|  | DBP | .059^e^ | .741 | .460 | .061 | .885 |
|  | RestHR | .020^e^ | .259 | .796 | .021 | .936 |
|  | RestSpO2 | .095^e^ | 1.183 | .239 | .097 | .872 |
|  | HR1 | .030^e^ | .386 | .700 | .032 | .933 |
|  | LFnu1 | -.007^e^ | -.085 | .932 | -.007 | .906 |
|  | HFnu1 | .035^e^ | .444 | .658 | .037 | .899 |
|  | Ratio1 | -.057^e^ | -.722 | .472 | -.060 | .893 |
|  | SDRR1 | .005^e^ | .063 | .950 | .005 | .780 |
|  | SD11 | -.012^e^ | -.149 | .881 | -.012 | .884 |
|  | SD21 | .018^e^ | .208 | .835 | .017 | .766 |
|  | nLFnu1 | .006^e^ | .076 | .939 | .006 | .976 |
|  | nHFnu1 | .027^e^ | .335 | .738 | .028 | .866 |
|  | nSD11 | -.004^e^ | -.050 | .960 | -.004 | .901 |
|  | nSD21 | .048^e^ | .588 | .558 | .049 | .826 |
|  | nSDRR1 | .031^e^ | .377 | .707 | .031 | .828 |
|  | deltaSpO21 | .006^e^ | .075 | .940 | .006 | .977 |
| a. Dependent Variable: DB1 | | | | | | |
| b. Predictors in the Model: (Constant), Caff | | | | | | |
| c. Predictors in the Model: (Constant), Caff, Exercise | | | | | | |
| d. Predictors in the Model: (Constant), Caff, Exercise, BR1 | | | | | | |
| e. Predictors in the Model: (Constant), Caff, Exercise, BR1, yearsgame | | | | | | |

| **Residuals Statistics^a^** | | | | | |
| --- | --- | --- | --- | --- | --- |
|  | Minimum | Maximum | Mean | Std. Deviation | N |
| Predicted Value | -56.9890 | 35.4519 | -17.2679 | 17.41208 | 153 |
| Residual | -183.24048 | 87.20964 | .08490 | 37.18522 | 153 |
| Std. Predicted Value | -2.275 | 3.036 | .007 | 1.000 | 153 |
| Std. Residual | -4.848 | 2.307 | .002 | .984 | 153 |
| a. Dependent Variable: DB1 | | | | | |
